# Síndrome Coronariana Aguda em um Jovem do Sexo Masculino com Uso Prolongado de Esteroides Androgênicos Anabolizantes

**DOI:** 10.36660/abc.20220233

**Published:** 2023-02-13

**Authors:** Daniel A. Gomes, Mariana Sousa Paiva, Sara Ranchordás, Rita Reis Santos, Jorge Ferreira, Marisa Trabulo

**Affiliations:** 1 Departamento de Cardiologia Hospital de Santa Cruz Carnaxide Portugal Departamento de Cardiologia, Hospital de Santa Cruz, Carnaxide – Portugal; 2 Departamento de Cirurgia Cardiotorácica Hospital de Santa Cruz Carnaxide Portugal Departamento de Cirurgia Cardiotorácica, Hospital de Santa Cruz, Carnaxide – Portugal

**Keywords:** Síndrome Coronariana Aguda, Aterosclerose, Receptores Androgênicos, Anabolizantes

## Introdução

Os esteroides androgênicos anabolizantes (EAA) são drogas sintéticas que mimetizam os efeitos da testosterona.^1^ O uso não médico de EAA ainda é um problema de saúde pública pouco reconhecido, mas relevante, e tem se tornado cada vez mais comum, especialmente em jovens do sexo masculino para fins físicos ou estéticos.^1 , 2^ Apesar de ter aplicabilidade no tratamento de algumas condições médicas, o abuso de EAA já foi associado à aterosclerose e à doença arterial coronariana (DAC) prematura.^1 - 4^

### Relato de caso

Um homem de 43 anos, sem história médica pregressa, deu entrada no pronto-socorro com dor torácica retroesternal iniciada no começo daquela manhã. Ele negou quaisquer sintomas acompanhantes. Os sinais vitais mostravam pressão arterial de 130/80mmHg, frequência cardíaca de 88bpm e saturação de oxigênio de 98%, e o exame físico foi normal, exceto por seu aspecto musculoso. Ele era fisiculturista e praticante de Muay Thai e relatava fazer uma dieta hiperproteica e hipercalórica. O paciente negou o uso de qualquer medicamento, bem como tabagismo ou etilismo. No entanto, quando questionado diretamente, ele relatou uso não-médico regular de EAA, incluindo nandrolona e testosterona intramuscular (1000mg a cada três meses) por mais de 20 anos. A história familiar era negativa para hipercolesterolemia ou doença cardiovascular.

**Figura 1:**
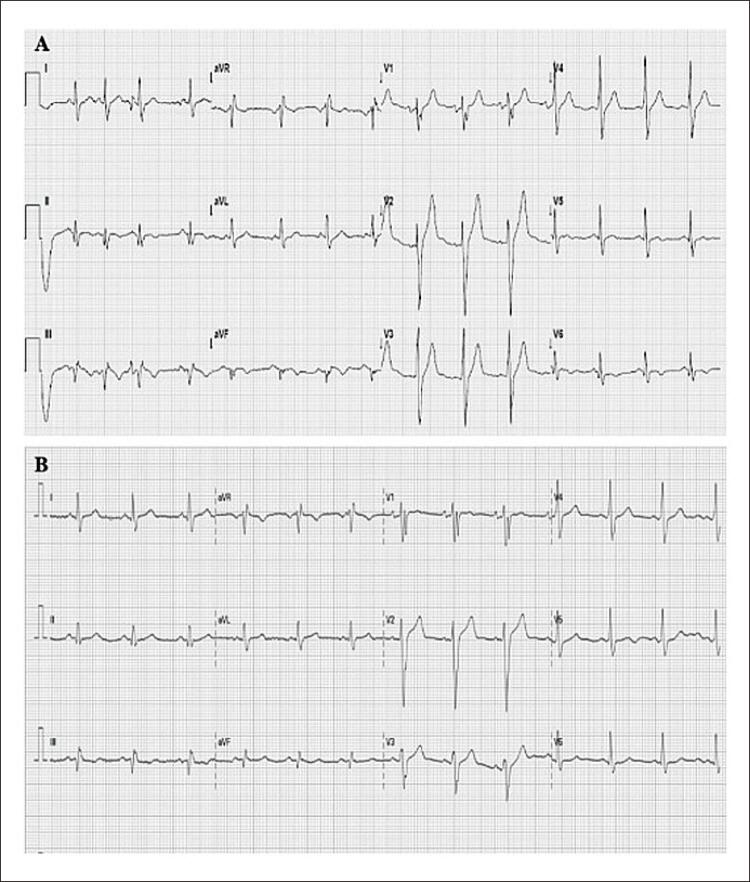
– Eletrocardiograma na hospitalização.

**Figura 2:**
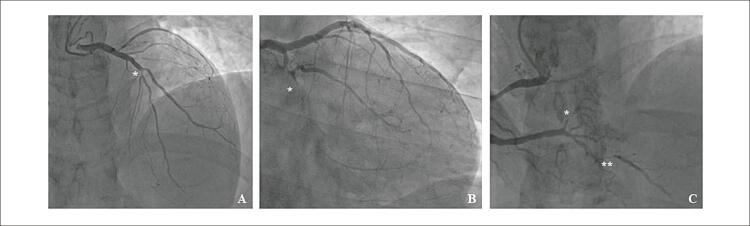
– Angiografia coronária. A) Lesão intermediária na ADA média na bifurcação com D1 (*). DA distal com lesões difusas. B) Oclusão total crônica da artéria circunflexa distal (*) com enchimento colateral retrógrado. C) Coronária direita dominante com lesões sub-oclusivas do ramo posterolateral (*culpado) e DP (**).

O eletrocardiograma de admissão revelou ritmo sinusal e distúrbios de condução intraventricular inespecíficos, sem desvios do segmento ST ( Figura 1 ). A Troponina-T cardíaca de alta sensibilidade chegou a 1224ng/L. O perfil lipídico em jejum mostrou lipoproteína de alta densidade-colesterol (HDL-C) significativamente baixo de 21mg/dL e níveis elevados de lipoproteína de baixa densidade-colesterol (LDL-C) de 229mg/dL. Os níveis de glicose e HbA1C estavam dentro da faixa de normalidade. O restante dos exames laboratoriais foi normal. Foi diagnosticado infarto agudo do miocárdio sem supradesnivelamento do segmento ST. Ao ecocardiograma transtorácico, a fração de ejeção do ventrículo esquerdo estava diminuída (FEVE de 39%) devido a hipocinesia difusa. Uma angiografia coronária invasiva revelou lesões suboclusivas do ramo posterolateral (culpado) e da artéria descendente posterior (DP), oclusão crônica total da artéria circunflexa distal e lesões intermediárias da artéria descendente anterior (DA) média esquerda e primeiro ramo diagonal (D1) ( Figura 2 ). O paciente foi submetido a cirurgia de revascularização do miocárdio (CRM) (artéria torácica interna (ATI) esquerda sequencial para D1 e DA, ATI direita como enxerto em T para o segundo ramo marginal obtuso e veia safena para DP).

A recuperação pós-operatória transcorreu sem intercorrências e ele recebeu alta após 5 dias com prescrição de betabloqueador, inibidor da enzima conversora de angiotensina, espironolactona, estatina de alta intensidade, terapia antiplaquetária dupla por um ano, seguida de aspirina vitalícia. O paciente foi orientado a seguir um estilo de vida saudável e aderir às medicações, e foi fortemente desaconselhado em relação ao uso de EAA.

Até o momento, ele permanece assintomático e livre de eventos cardiovasculares. Uma ressonância magnética cardíaca (RMC) aos 12 meses de seguimento revelou disfunção e dilatação biventricular moderada (FEVE 37%, FEVD 38%) sem defeitos de perfusão induzidos por estresse. Apesar de todas as recomendações, o paciente ainda mantém consumo regular de corticoide e relata não adesão à terapia com estatina.

## Discussão

Existem apenas alguns relatos de casos de síndromes coronarianas agudas relacionadas a EAA em indivíduos jovens, a maioria relacionada à ingestão prolongada de altas doses de EAA e dislipidemia grave.^3^ Embora as evidências disponíveis sejam visivelmente escassas, estudos anteriores sugerem que o abuso de EAA está associado à aterosclerose coronariana acelerada e eventos cardiovasculares.^1 , 2^

Os mecanismos pelos quais os EAA podem promover DAC são diversos e incluem aterogenicidade, trombogenicidade e reatividade vascular.^1 , 4^ O uso de EAA induz uma modificação aterogênica do perfil lipídico, ao reduzir consistentemente o HDL-colesterol e, em menor grau, aumentar os níveis do LDL-colesterol.^5^ Também se constatou que os EAA promovem um estado pró-trombótico, principalmente pelo aumento da ativação e agregação plaquetária.^1 , 3^

Este caso é ilustrativo dos efeitos deletérios dos EAA no sistema cardiovascular. Ele relata a ocorrência de infarto agudo do miocárdio (IAM) em um usuário de EAA com dislipidemia grave e carga aterosclerótica coronariana. O abuso de EAA pode ser considerado um importante fator predisponente, ao promover o desenvolvimento de dislipidemia grave. De fato, as alterações observadas no perfil lipídico desse paciente (níveis notavelmente baixos de HDL-C e altos de LDL-C) são consistentes com relatos anteriores de doença arterial coronariana e IAM relacionada a EAA.^3^ Juntamente com o consumo de EAA, hábitos alimentares não saudáveis também podem ter contribuído para a aterogenicidade. Embora não possamos excluir uma predisposição individual à aterosclerose através de testes genéticos, não havia história familiar de hipercolesterolemia ou doença arterial coronariana prematura.

O fato de o paciente manter o consumo de corticosteroides e apresentar disfunção biventricular um ano após a revascularização completa – e sem isquemia residual na RMC de estresse – levanta a possibilidade de cardiomiopatia associada ao EAA. De fato, estudos anteriores demonstraram o papel do EAA isoladamente no desenvolvimento de comprometimento reversível do miocárdio e cardiomiopatia dilatada.^6^ Uma RMC realizada após cessação do consumo de EAA seria de interesse para confirmar esta hipótese.

Em conclusão, o abuso de EAA, ainda pouco reconhecido, deve sempre ser considerado como um potencial fator de risco modificável e predisponente em indivíduos jovens com síndrome coronariana aguda.
