# Supplementary material for: Síndrome Coronariana Aguda em um Jovem do Sexo Masculino com Uso Prolongado de Esteroides Androgênicos Anabolizantes
Source: Arq Bras Cardiol. 2023 Feb 13;120(2):e20220233. [Article in Portuguese] doi: 10.36660/abc.20220233 (PMC9972683; doi:10.36660/abc.20220233)
Supplement: Translation

# Translation: Acute Coronary Syndrome in a Young Male with Long-Term Use of Anabolic-Androgenic Steroids

**Authors:** Daniel A. Gomes, Mariana Sousa Paiva, Sara Ranchordás, Rita Reis Santos, Jorge Ferreira, Marisa Trabulo

**Affiliations:** 1 Portugal Departamento de Cardiologia, Hospital de Santa Cruz, Carnaxide – Portugal; 2 Portugal Departamento de Cirurgia Cardiotorácica, Hospital de Santa Cruz, Carnaxide – Portugal

**Keywords:** Acute Coronary Syndrome, Atherosclerosis, Androgen Receptors, Anabolic Agents

## Introduction

Anabolic-androgenic steroids (AAS) are synthetic drugs that mimic the effects of testosterone.^1^Non-medical use of AAS is still an underrecognized but relevant public health issue and it has become increasingly common, especially in young males for physical performance or aesthetic purposes.^1 , 2^Despite having applicability in treating some medical conditions, AAS abuse was previously linked to atherosclerosis and premature coronary artery disease (CAD).^1 - 4^

### Case report

A 43-year-old man, without a past medical history, was admitted in the emergency room with retrosternal chest pain starting earlier that morning. He denied any accompanying symptoms. The vital signs showed a blood pressure of 130/80mmHg, heart rate of 88bpm and oxygen saturation of 98%, and physical examination was unremarkable except for his muscular appearance. He was a bodybuilder and *Muay Thai* practitioner and reported eating a hyperproteic and hypercaloric diet. The patient denied taking any medication, as well as smoking or alcohol consumption. However, when asked directly, he reported regular non-medical use of AAS, including intramuscular nandrolone and testosterone (1000mg every three months) for more than 20 years. Family history was negative for hypercholesterolemia or cardiovascular disease.

The admission electrocardiogram revealed sinus rhythm and nonspecific intraventricular conduction disturbances, without ST segment deviations ( Figure 1 ). High-sensitivity cardiac troponin-T was up to 1224ng/L. Fasting lipid profile showed significantly low high-density lipoprotein-cholesterol (HDL-C) of 21mg/dL and high levels of low-density lipoprotein-cholesterol (LDL-C) of 229mg/dL. Glucose and HbA1C levels were within the normal range. The remaining laboratory workup was normal. Non-ST segment elevation acute myocardial infarction was diagnosed. On the transthoracic echocardiography, the left ventricular ejection fraction was reduced (LVEF of 39%) due to diffuse hypokinesia. An invasive coronary angiography revealed sub-occlusive lesions of the posterolateral branch (culprit) and of the posterior descending artery (PD), chronic total occlusion of the distal circumflex artery, and intermediate lesions of the mid left anterior descending artery (LAD) and first diagonal branch (D1) ( Figure 2 ). The patient was submitted to a coronary artery bypass graft (CABG) surgery (left internal thoracic artery (ITA) sequential to D1 and LAD, right ITA as a T-graft to the second obtuse marginal branch and saphenous vein to PD).

**Figure 1:** – Electrocardiogram at hospital admission.

**Figure 2:** – Coronary angiography. A) Intermediate lesion in the mid LAD at the bifurcation with D1 (*). Distal LAD with diffuse lesions. B) Chronic total occlusion of the distal circumflex artery (*) with retrograde collateral filling. C) Dominant right coronary artery with sub-occlusive lesions of the posterolateral branch (* culprit) and PD (**).

Postoperative recovery was uneventful, and he was discharged after 5 days with a prescription of beta-blocker, angiotensin-converting enzyme inhibitor, spironolactone, high-intensity statin, dual antiplatelet therapy for 1 year followed by lifelong aspirin. The patient was recommended to comply with a healthy lifestyle and medication, and strongly advised against the use of AAS.

To date, he remains asymptomatic and free of cardiovascular events. A cardiac magnetic resonance imaging (CMR) at 12 months of follow-up revealed moderate biventricular disfunction and dilatation (LVEF 37%, RVEF 38%) without any stress-induced perfusion defects. Despite all the recommendations, the patient still maintains regular steroid consumption and reports not adhering to statin therapy.

## Discussion

There are only a few case reports of AAS-related acute coronary syndromes in young individuals, most related to long-term intake of high doses of AAS and severe dyslipidemia.^3^Although the available evidence is noticeably scarce, previous studies suggest that AAS abuse is associated with accelerated coronary atherosclerosis and cardiovascular events.^1 , 2^

The mechanisms by which AAS may promote CAD are diverse and include atherogenicity, thrombogenicity and vascular reactivity.^1 , 4^AAS use induces an atherogenic modification of the lipid profile, by consistently lowering HDL cholesterol and, to a lesser extent, increasing LDL cholesterol levels.^5^AAS have also been consistently found to promote a pro-thrombotic state, mainly by increasing platelet activation and aggregation.^1 , 3^

This case is illustrative of the deleterious effects of the AAS on the cardiovascular system. It reports an acute myocardial infarction (AMI) presenting in a AAS user with severe dyslipidemia and coronary atherosclerotic burden. AAS abuse could be considered a major predisposing factor, by promoting the development of severe dyslipidemia. In fact, the observed changes in this patient’s lipidic profile (remarkably low HDL-C levels and high LDL-C) are consistent with previous reports of AAS-related coronary artery disease and AMI.^3^Along with AAS consumption, unhealthy dietary habits may have also contributed to the atherogenicity. Even though we cannot exclude an individual predisposition to atherosclerosis by means of genetic testing, there was no family history of hypercholesterolemia or premature coronary artery disease.

The fact that the patient maintains steroids consumption and presents with biventricular dysfunction one year after complete revascularization – and without residual ischemia in the stress CMR – raises the possibility of associated AAS-related cardiomyopathy. In fact, previous studies have shown the role of AAS alone in the development of reversible myocardial impairment and dilated cardiomyopathy.^6^A CMR performed after cessation of AAS consumption would be of interest to confirm this hypothesis.

In conclusion, AAS abuse, still largely underrecognized, should always be considered as a potential predisposing and modifiable risk factor in young subjects presenting with an acute coronary syndrome.
